# Molecular Basis of Cisplatin Resistance in Testicular Germ Cell Tumors

**DOI:** 10.3390/cancers11091316

**Published:** 2019-09-06

**Authors:** Violeta Bakardjieva-Mihaylova, Karolina Skvarova Kramarzova, Martina Slamova, Michael Svaton, Katerina Rejlova, Marketa Zaliova, Alena Dobiasova, Karel Fiser, Jan Stuchly, Marek Grega, Blanka Rosova, Roman Zachoval, Petr Klezl, Vaclav Eis, Eva Kindlova, Tomas Buchler, Jan Trka, Ludmila Boublikova

**Affiliations:** 1 Department of Pediatric Hematology and Oncology, CLIP, 2nd Faculty of Medicine, Charles University and University Hospital Motol, 150 00 Prague, Czech Republic (V.B.-M.) (K.S.K.) (M.S.) (M.S.) (K.R.) (M.Z.) (A.D.) (K.F.) (J.S.) (J.T.); 2 Department of Pathology and Molecular Medicine, 2nd Faculty of Medicine, Charles University and University Hospital Motol, 150 00 Prague, Czech Republic; 3 Department of Pathology and Molecular Medicine, 3rd Faculty of Medicine, Charles University and Thomayer Hospital, 140 00 Prague, Czech Republic; 4 Department of Urology, 3rd Faculty of Medicine, Charles University and Thomayer Hospital, 140 00 Prague, Czech Republic; 5 Department of Urology, 3rd Faculty of Medicine, Charles University and University Hospital Kralovske Vinohrady, 100 00 Prague, Czech Republic; 6 Department of Pathology, 3rd Faculty of Medicine, Charles University and University Hospital Kralovske Vinohrady, 100 00 Prague, Czech Republic; 7 Department of Radiotherapy and Oncology, 3rd Faculty of Medicine, Charles University and University Hospital Kralovske Vinohrady, 100 00 Prague, Czech Republic; 8 Department of Oncology, 1st Faculty of Medicine, Charles University and Thomayer Hospital, 140 00 Prague, Czech Republic

**Keywords:** cisplatin resistance, testicular germ cell tumor, molecular aberrations, next generation sequencing, cell cycle

## Abstract

The emergence of cisplatin (CDDP) resistance is the main cause of treatment failure and death in patients with testicular germ cell tumors (TGCT), but its biologic background is poorly understood. To study the molecular basis of CDDP resistance in TGCT we prepared and sequenced CDDP-exposed TGCT cell lines as well as 31 primary patients’ samples. Long-term exposure to CDDP increased the CDDP resistance 10 times in the NCCIT cell line, while no major resistance was achieved in Tera-2. Development of CDDP resistance was accompanied by changes in the cell cycle (increase in G1 and decrease in S-fraction), increased number of acquired mutations, of which 3 were present within ATRX gene, as well as changes in gene expression pattern. Copy number variation analysis showed, apart from obligatory gain of 12p, several other large-scale gains (chr 1, 17, 20, 21) and losses (chr X), with additional more CNVs found in CDDP-resistant cells (e.g., further losses on chr 1, 4, 18, and gain on chr 8). In the patients’ samples, those who developed CDDP resistance and died of TGCT (2/31) showed high numbers of acquired aberrations, both SNPs and CNVs, and harbored mutations in genes potentially relevant to TGCT development (e.g., *TRERF1*, *TFAP2C* in one patient, *MAP2K1* and *NSD1* in another one). Among all primary tumor samples, the most commonly mutated gene was *NSD1*, affected in 9/31 patients. This gene encoding histone methyl transferase was also downregulated and identified among the 50 most differentially expressed genes in CDDP-resistant NCCIT cell line. Interestingly, 2/31 TGCT patients harbored mutations in the *ATRX* gene encoding a chromatin modifier that has been shown to have a critical function in sexual differentiation. Our research newly highlights its probable involvement also in testicular tumors. Both findings support the emerging role of altered epigenetic gene regulation in TGCT and CDDP resistance development.

## 1. Introduction

Testicular germ cell tumors (TGCT) are the most frequent solid tumors in young adult men aged 18–45 years. The incidence of TGCT has increased over the past decades, but the reason for this observation is unknown [1,2,3,4,5]. Although TGCT possess very pronounced hereditary patterns with sons and brothers of patients having increased risk of this malignancy (4–6 fold and 8–10 fold, respectively), no single major genetic aberration has been linked to TGCT development but an increasing number of low susceptibility risk loci have been described in the affected families and from genome-wide association studies [6,7,8,9,10]. The genetic hallmark of invasive TGCT is the amplification of the short arm of chromosome 12, usually in the form of isochromosome i(12p), which may lead to the activation of genes located here, most importantly the core stem cell gene *NANOG* or the oncogene *KRAS*. Another typical alteration is the KIT/KITL pathway activation, characteristic for early stages and pre-invasive tumors [11,12,13,14,15]. In general, TGCT harbor low numbers of somatic mutations, having low tumor mutation burden (TMB) in comparison with other adult solid tumors (and resembling rather childhood malignancies) but poses more complex genetic abnormalities, such as larger rearrangements, ploidy changes, epigenetic alterations, etc. [10,16]. Moreover, the interactions of germ cells with an altered interstitial microenvironment, including Sertoli and Leydig cells that influence germ cell growth and differentiation, represent another important aspect of TGCT development. All these factors may combine in their effect, leading to the critical event in TGCT formation—the abnormal cell division leading to aneuploidy and copy number variations, regularly found in this type of tumor [16,17].

Based on the characteristic histological patterns, TGCT are classified into seminomas and nonseminomas. These two main TGCT subtypes show distinct clinical characteristics, differing in the course of the disease and treatment response. Introducing platinum-based regimens into the treatment of TGCT dramatically increased the overall cure rate, with over 90% of patients remaining in long-term complete remission [18,19]. Nevertheless, there is currently no effective treatment for the platinum-refractory patients. There have been attempts to introduce the targeted therapy into the management of resistant TGCT (such as small molecular inhibitors—e.g., imatinib, an inhibitor of the KIT/KITL signaling pathway; or monoclonal antibodies—e.g., bevacizumab, an inhibitor of VEGF (vascular endothelial growth factor), angiogenesis, and tumor growth, recently also immune check-point inhibitors), but the results of these studies were unsatisfactory [20,21,22,23,24].

The principal cause of the development of cisplatin (CDDP) resistance, which almost inevitably leads to the treatment failure and death of the affected TGCT patients, has not been identified, and the molecular basis of cisplatin resistance is poorly understood. In general, the resistance to cisplatin may arise at any phase of its kinetics and action [25]. However, in case of TGCT, the basis of CDDP resistance is quite different from that seen in other solid tumors of adults, and means rather the loss of the exceptionally high sensitivity to the platinum-based chemotherapy, which is a typical feature of TGCT. This high sensitivity to CDDP is supposed to be related to the germ and embryonic character of the tumor cells and so called embryonic type of hypersensitive DNA damage response (DDR). In the cells with activated/overexpressed core stem cell genes (*NANOG*, *OCT3/4/POU5F1*), the cytotoxic stress/DNA damage drives these cells via p53 signaling directly to apoptosis, not allowing for any attempts of damage repair. The loss of this specific type of DDR, e.g. accompanying the differentiation of tumor cells, either spontaneous or induced by the applied chemotherapy, may then result in the establishment of the CDDP resistance [26,27,28,29]. Indeed, the aberrations that have been so far identified to occur with higher frequency in CDDP-resistant TGCT patients include the mutations in *TP53* and *MDM2* genes [29,30]. In further, recent and more experimental studies, CDDP resistance was also related to aberrations of DNA-repair genes and regulators (*XRCC2*, *HMGB4*) [31,32], oncogenes (*PIK3CA*, *IGF1R*) [31,33], cell cycle check point disruptors (*MAD2g*) [34] or epigenetic remodeling complexes (PRC1/2, namely *BMI1*) [35].

In this project, we generated cisplatin-exposed counterparts of TGCT cell lines and investigated their newly acquired features in comparison with the original cells, to uncover particular genes or pathways involved in the acquired cisplatin resistance. In a group of primary tumor samples of TGCT patients with different stages of the disease, we then looked for similar aberrations and for aberrations that could be related to the disease development.

## 2. Results

### 2.1. Establishment and Characterization of Cisplatin-Resistant TGCT Cell Lines

#### 2.1.1. Resistance to Cisplatin

**Figure 1:**
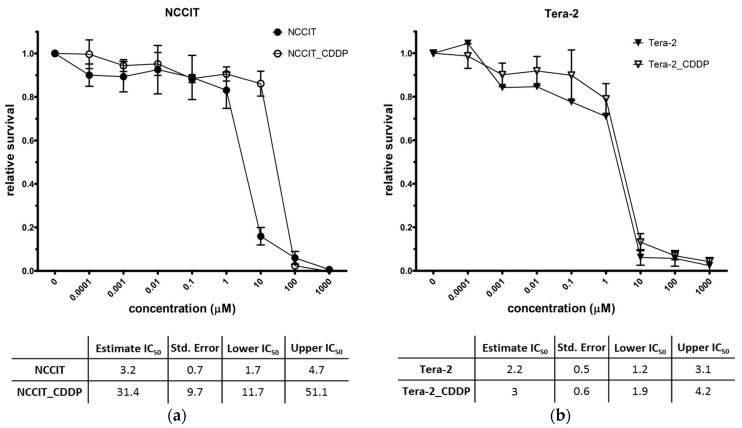
Final sensitivity of TGCTs cell lines to CDDP measured by MTS assays after 72 h of CDDP treatment: (**a**) CDDP-naive (NCCIT) and CDDP-treated (NCCIT_CDDP) NCCIT cell lines; (**b**) CDDP-naive (Tera-2) and CDDP-treated (Tera-2_CDDP) Tera-2 cell lines (the means and SDs of 4 independent assays for each cell line are displayed, each assay analyzed in triplicates or 6-plicates).

NCCIT cell line was cultured with increasing doses of CDDP for 20 months (further referred to as NCCIT_CDDP), achieving a significant resistance to CDDP, with IC_50_ approximately 10 times higher than the original sensitive cells (referred to as NCCIT) (NCCIT IC_50_ = 3.2 µM, NCCIT_CDDP IC_50_ = 31.4 µM; with nonoverlapping confidence intervals; Figure 1a). Tera-2 cell line was handled similarly, but no major escalation of CDDP concentration was possible due to its persisting high sensitivity even after 15 months of CDDP treatment (initial CDDP IC_50_ = 2.2 µM, final IC_50_ = 3 µM; Figure 1b). No significant increase in IC_50_ could be achieved, and repeated attempts of CDDP concentration escalation had a lethal effect on Tera-2 cells (original cell line referred to as Tera-2, cell line cultivated long-term with CDDP referred to as Tera-2_CDDP).

#### 2.1.2. Cell Cycle and Proliferation

**Figure 2:**
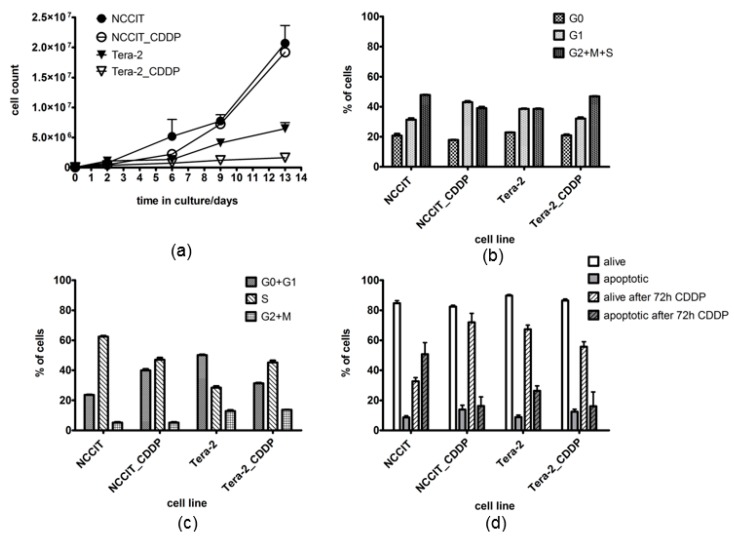
Proliferation and cell cycle of TGCTs cell lines. (**a**) proliferation of the cells expressed in total numbers of cells during two week cultivation; (**b**) pyronin/hoechst cell cycle analysis; (**c**) EdU cell cycle analysis; (**d**) apoptosis analysis without CDDP treatment and after 72 h of CDDP treatment.

There was a notable difference in the proliferation between the two original cell lines, NCCIT showing much faster proliferation rate than Tera-2. The derived counterparts of both cell lines showed a slower proliferation rate compared with that of the parental sensitive cells, which was nonsignificant and only transient in case of NCCIT_CDDP (around day 6), but profound and permanent in case of Tera-2_CDDP (Figure 2a). To further examine the observed differences in cell growth, we analyzed the cell cycle and apoptotic rate of the cells. There were more of the resistant NCCIT_CDDP cells in G1 phase when compared with the parental NCCIT cell line (43 vs. 31%; Figure 2b, *p* = 0.05), which was accompanied by a decreased number of resistant NCCIT_CDDP cells in S phase (47% vs. 63%; Figure 2c, *p* = 0.01). Interestingly, the trend in the cell cycle distribution between parental vs. derived Tera-2 cells was rather opposite. Tera-2_CDDP showed a decrease in G1 (39% vs. 32%; Figure 2b, *p* > 0.05) and an increase in S phase in comparison with parental cell line (29% vs. 45%; Figure 2c, *p* = 0.05). The apoptotic rate of the cell lines was analyzed before and after a short exposure to cisplatin. Apoptosis analysis in the absence of CDDP indicated a good and consistent viability of all analyzed cell lines regardless of their different proliferation rate (Figure 2d). After 72 hours of CDDP treatment (10 µM), the resistant NCCIT_CDDP cells showed almost no decrease in viability, while the sensitive NCCIT showed increased apoptosis (51% compared with 9%; *p* = 0.05) and a dramatic drop in the number of alive cells (33% vs. 83%; Figure 2d, *p* = 0.03). In Tera-2 cell lines, mild increase in apoptotic response was also observed in the original CDDP-naive cells, and no change was observed in CDDP-exposed cells after incubation with CDDP.

### 2.2. Molecular Genetics Studies

#### 2.2.1. Molecular Genetic Profile of TGCT Cell Lines

Comparisons of whole exome sequencing (WES) data of both original and derived TGCT cell lines showed a significantly increased total number of acquired gene variants in the cells that developed CDDP resistance (the acquired variant determined as a significant gene variant present in at least 80% of sequencing reads of CDDP-treated cells and not present in the original cell line and the long-term co-cultivated control—see methods in the Supplementary Material for details; Table 1). In resistant NCCIT_CDDP cells, 21 acquired missense or frameshift variants were identified, while in the case of Tera-2_CDDP cells, that did not develop CDDP resistance, only one acquired missense variant was found in *OPN1LW* gene (present in 91% of sequencing reads). Among the acquired variants found in NCCIT_CDDP cells we identified genes involved in cellular pathways crucial for survival, proliferation, metastatic dissemination as well as metabolic set up of cancer cells – e.g., genes involved in DNA damage repair (*BCOR*), chromatin remodeling (*BRWD3*, *ATRX*), major signaling pathways (*MAP3K4*, *COMP*), metabolic pathways (*SLC22A2*, *HPSE*) or maintenance of epithelial-mesenchymal balance (*KDR*). Interestingly, we found three different missense variants in *ATRX* gene acquired during the development of CDDP resistance (two of them present in 100% and one in 95% of sequencing reads of the sequenced NCCIT_CDDP cell line; Table 1).

When analyzing the number of variants in different signaling pathways (as defined by Kegg Pathway database [36]), there was an increase in variants in the cell cycle pathway and proteasome pathway in resistant NCCIT_CDDP cells comparing with the other cell lines;, and the number of variants in the steroid hormone biosynthesis pathway was high in both original NCCIT and resistant NCCIT_CDDP cells in comparison with Tera-2 or Tera-2_CDDP cells (Supplementary Figure S1).

**Figure 3:**
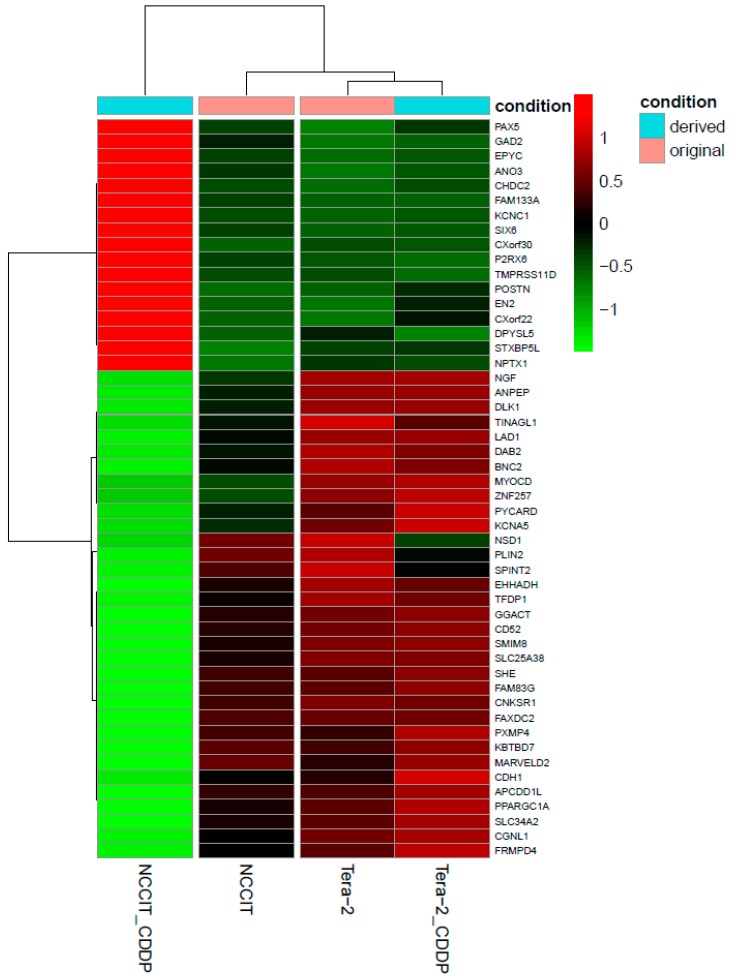
Hierarchical clustering of original and CDDP-treated cell lines based on gene expression profiles of the top 50 differentially expressed genes.

From transcriptome sequencing, a set of 50 most differentially expressed genes between CDDP-resistant (CDDP_NCCIT) and sensitive cell lines (NCCIT and both CDDP_Tera-2 and Tera-2) was identified (Figure 3). Unsupervised hierarchical clustering of the genes showed substantial differences in gene expression between original and derived NCCIT cell lines. Among these, we found additional cancer-related genes e.g., transcription factors such as *PAX5*, *DAB2*, *SPINT2*, *TFDP1*, histon methyltransferase *NSD1*, as well as other genes previously described to regulate proliferation (*DLK1*), cell signaling (*CNKSR1*, *CNFSR1*, *APCDD1*, *EPYC*), metabolism (*GAD2*, *ANPAP*, *PPARGC1A*), cell migration and adhesion (*CDH1*, *POSTN*). Altered expression was also present in *PYCARD* gene, which is a key mediator in apoptosis and inflammation. We also detected alteration in the expression of two homeobox genes (*SIX6* and *EN2*).

No newly acquired gene fusion has been detected in relation to developed CDDP resistance. In both original and derived NCCIT cell lines we found two in-frame fusion transcripts, *SBF2/RNF141* and *EML4/MTA3*, resulting from an in-frame deletion and tandem duplication, respectively. Their presence was also confirmed by Sanger sequencing. In the case of the Tera-2 cell line, three in-frame fusion transcripts were found in both the original and derived counterparts. Two of them (*SPG7/CDH15* and *ZNHIT6/COL24A*) originated from tandem duplication in the coding sequence, while in *ZNF160/ZNF415* the coding sequence of one gene was translocated to the 5′ untranslated region of the second gene (Supplementary Table S1).

**Figure 4:**
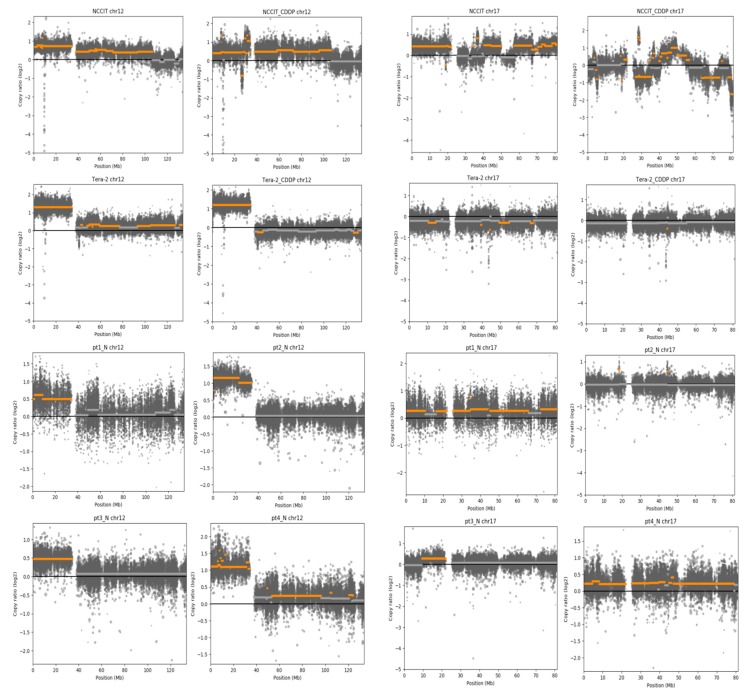
Scattered plots displaying copy number variation ratios inferred from WES data normalized to pooled control samples. Segmental changes with predicted copy number alteration are marked in orange, normal in gray. Chromosomes 12 and 17 as the most clinically relevant displayed here, all are in the supplementary material (Supplementary Figure S2).

Copy number variation (CNV) analysis revealed large-scale gains and losses in all cell lines. Apart from the obligatory 12p gain, characteristic of TGCT, other CNVs could be identified particularly on chromosome 1, 20, 21 and X. Further acquired CNVs changes were observed in the NCCIT_CDDP cell line comparing with the original NCCIT counterpart (on chromosomes 1, 4, 8, 9, 10, 13, 17, 18 and X) (Figure 4 and Supplementary Figure S2).

Tumor mutation burden (TMB) was slightly higher in the CDDP-derived cells than in the original cell lines and did not differ between NCCIT and Tera-2 cell lines (TMB ratio derived/original cell line 1.09 and 1.06 for NCCIT and Tera-2, respectively; Table 2).

#### 2.2.2. Molecular Genetic Profile of TGCT Patients

In order to compare the genetic variants found in the TGCT cell lines with patients samples, we analyzed primary tumor samples by WES (four samples) or amplicon sequencing with the Comprehensive Cancer panel (over 400 genes; 27 samples), together with patients’ germline DNA as a background control. 

In 3 out of 4 patients analyzed in detail by WES, significant acquired somatic gene aberrations were found (Table 3). The patient who subsequently died of the disease progression (Pt2) harbored 6 missense variants, including the mutation present with the highest variant frequency (45%) in *TFAP2C* gene, and another clinically interesting mutation with high variant frequency (30%) within *TRERF1* gene. Of the other two patients, one harbored a stop/gain mutation in *DOHH* gene—metalloenzyme involved in lysine metabolism; and the other was detected with 7 acquired missense variants including genes *RIN1*—member of RAS signaling pathway, *FOXG1*—member of FOXO signaling pathway, and DMRTA1—transcription factor. There was no overlap in the affected genes among these 3 patients.

**Figure 5:**
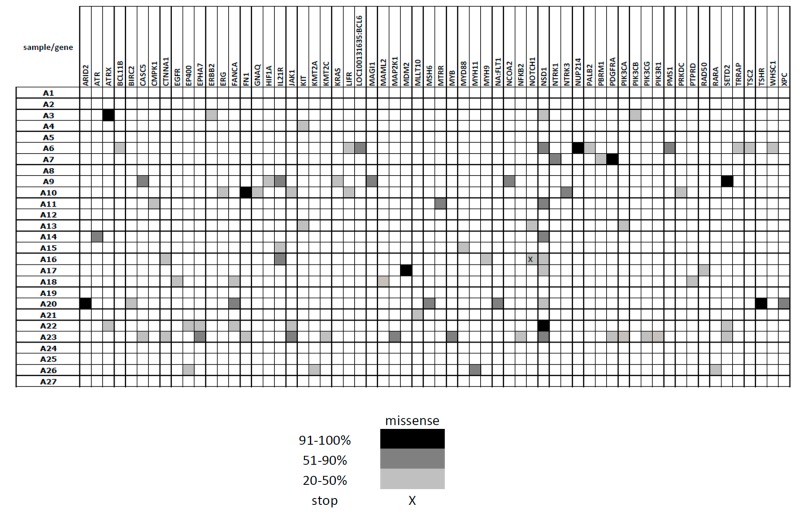
Somatic variants detected by amplicon sequencing in primary TGCT tumors (in comparison to germ-line control patient samples)—variants present in at least 20% of sequencing reads.

In the larger group of patients who underwent amplicon sequencing, a number of gene aberrations were detected, some of the genes being affected in multiple patients (Figure 5). The most commonly affected gene was *NSD1*, encoding histone methyltransferase, that was altered in 9 of 27 (33%) patients. Interestingly, we also found variations in the *SETD2* gene, another histone methyltransferase, in 3 of 27 (11%) patients. Variations in 3 other genes—*FANCA*, *IL21R*, and *JAK1* were present in more than two patients. The genes with the highest variant frequency (91–100%, indicating their presence in homozygous status) included *ATRX*, *ARID2*, *FANCA*, *MDM2*, *PDGFRA*, *NSD1*, *SETD2*, and *TSHR.* The highest number of altered genes (15) was found in the only one patient of this cohort who died of the disease progression. The only gene that was found to be altered in relation to CDDP resistance in TGCT cell lines and was present also in TGCT patients was *ATRX* gene (2 patients, 7%).

The comparison with clinical and laboratory data did not prove any relation between the number of detected variants and the histologic subtype (seminoma vs. nonseminoma) but there was a trend towards increasing number of variations in advanced stages of the tumor (Supplementary Figure S3).

The WES-sequenced patient samples were also analyzed for CNV and TMB. Apart from the expected 12p gain, we could observe large scale gains and losses on other chromosomes—e.g., gains on chromosome 17 in 2/4 pts (Figure 4 and Supplementary Figure S4). The one patient, who had CDDP-resistant tumor and died of TGCT (pt2), had losses on chromosomes 1, 4, 9, 18 and gains on chromosome 8—a similar pattern as observed in case of CDDP-resistant NCCIT cell line. He had also the highest TMB ratio (over 4 when related to the germ-line control). In the remaining 3 patients we also observed increased mutation burden in tumor samples in comparison to the controls, but not as pronounced (1.69–2.88; Table 4).

## 3. Discussion

The topic of cisplatin resistance is highly relevant as it represents the leading cause of treatment failure in TGCT management. No single major aberration has been so far associated with CDDP resistance in TGCT but increasing number of potential genes, pathways, genomic and epigenetic changes have been emerging from recent experimental and descriptive studies. In order to add further information to the understanding of the basis and conditions leading to cisplatin resistance development, we established two CDDP-exposed cell lines and studied their proliferative and genetic properties. NCCIT and Tera-2 are both non-seminoma TGCT cell lines that contain embryonic carcinoma cells. NCCIT is referred to include also a teratocarcinoma component. Both cell lines showed similar initial sensitivity to CDDP. With the identical approach of intermittent CDDP exposure and resurrection periods, NCCIT developed a significant CDDP resistance (10 times higher in comparison with the original cell line, similar to the previously published studies [24,37]) while Tera-2 kept its sensitivity and no significant increase in CDDP resistance could be achieved. While high chemosensitivity is typical for embryonic carcinoma cells and is associated with the embryonic features these tumor cells keep, teratocarcinoma represents a more differentiated variant of TGCT and even in the clinical practice this TGCT subtype is characterized by high chemotherapy resistance.

It is well known that cytotoxic chemotherapy is not effective for cancer cells in the G0/G1 phase, and resistant cancer cells are prone to rest at this stage [38,39,40]. This situation was observed in the cell line which developed the resistance to cisplatin. In these NCCIT_CDDP cells, there was about a 10% increase of cells present in the G0/G1 phase of the cell cycle, with a significant decrease in the number of cells in the S phase. The CDDP-resistant cells also showed no increase in apoptosis after CDDP exposition, contrary to the remaining cell lines. This suggests the switch in the specific DNA-damage response (DDR) signaling from the embryonic type, typical for TGCT (primary activation of apoptotic pathway), to that common for other solid tumors (activation of cell cycle control and DNA repair pathways). The initial higher proliferation rate of the NCCIT cell line may also facilitate the development of CDDP resistance by faster accumulation of potentially functional mutations and faster selection of the resistant cells.

The DNA and RNA sequencing revealed two specific gene aberrations that may have a strong functional relation to TGCT and CDDP resistance development – the mutations in *ATRX* gene and the alterations of *NSD1* gene. *ATRX* was the gene with the highest frequency of newly acquired mutations found in the CDDP-resistant cell line and the only one mutated also in primary TGCT tumors. This gene is located on chromosome Xq21.1 and encodes an α-thalassemia mental retardation X-linked protein, a member of the SWI/SNF superfamily of chromatin modifiers. It regulates various cellular processes such as replication stress response, transcription or mitotic recombination, and has been shown to play a critical role in sexual differentiation [41]. Aberrations within *ATRX* sequence or alterations of its expression were found in various cancers including glioma, neuroblastoma, osteosarcoma, colorectal cancer or lung cancer [42]. They have not been reported yet in relation to TGCT; but as the ATRX syndrome, caused by *ATRX* mutations, involves apart from thalassemia and mental retardation also gonadal dysgenesis of various degree, in extreme cases even male to female sex reversal [43], and as the testicular dysgenesis syndrome is the common precursor condition in TGCT, it supports its probable functional role in TGCT development.

Mutations of *NSD1* gene were the most common aberrations detected in TGCT patients in this study including one with CDDP-resistant disease. The other CDDP-resistant patient had large scale losses including this gene region on chromosome 5, and downregulation of this gene was also found within the 50 most differentially expressed genes in CDDP resistant cell line comparing to the sensitive cells. *NSD1* is located on chromosome 5q35.3, encodes nuclear receptor binding SET domain protein 1, which functions as the methyltransferase H3K36. It has been reported as an oncogene or tumor suppressor depending on cellular context; and its aberrant expression or mutations have been described previously [44,45]. Alterations of *NSD1* are strongly associated with widespread genome hypomethylation [46]. It is implicated in the early prenatal development, enhances androgen receptor transactivation, and has been related to Sotos syndrome, presenting mostly with growth and neurological abnormalities, but sometimes also with cryptorchidism and malignant tumors [47].

The fact that both altered genes *ATRX* and *NSD1* are involved in epigenetic regulations, DNA methylation and chromatin remodeling correlates well with the current understanding to TGCT biological background, where larger genomic and epigenomic aberrations rather than point mutations are supposed to represent the crucial events in TGCT pathogenesis. In accordance with this, repression of H3K27 methylation was associated with acquired CDDP resistance in TGCT in one recent study [35].

Of the other genes found altered in primary TGCT tumors in this study, some have been related to TGCT previously. These include genes related to TGCT occurrence in published genome wide association studies (GWAS) or other genetic association studies, such as *FOXG1* [48], *OPN1LW* [49], *EOMES* [50,51], *TFAP2C* [52], *DMRTA1* [6,53,54]; and genes related to CDDP resistance in TGCT patients—like *MDM2*, which is the only one gene that have been related to CDDP resistance in TGCT patients repeatedly [30,31,55,56]. We found this gene mutated (in homozygous state—almost 100% of reads) in one primary TGCT sample but not in the resistant ones.

In the primary tumor samples we also identified mutations of several genes that have not been associated with TGCT so far but which role in TGCT could be implied. These include e.g., *SETD2* and *FANCA* genes. *SETD2* gene, whose variants were found in 10% of patients in our cohort, is another gene with the influence on epigenetic remodeling. It is an important partner of *NSD1* gene, acts as a tumor suppressor [57,58,59,60] and a key regulator of genome stability [61]. Like *NSD1*, it has not been described in relation to TGCT, but it seems to be involved in spermatogenesis and its aberrations may be related to male infertility [62]. *FANCA* gene mutations were also present in 10% of TGCT patients; while this gene is well known for its function in post-replication DNA repair and maintenance of chromosome stability, its high expression levels were found in pachytene spermatocytes (in mice) and its role in the maintenance of reproductive germ cells and in meiotic recombination is suggested [63].

CDDP resistance was in our study associated with aberrations in genes *MAP2K1*, *NSD1*, *TRERF1* and *TFAP2C* in resistant primary TGCT and several other genes, namely *KDR* and *MAP3K4* in the resistant cell line. *TFAP2C* gene encodes a sequence-specific DNA-binding transcription factor, which is involved in activation of several developmental genes. It has a significant role in the development of primordial germ cells, and its altered expression was found in some cancer types, including murine germ cell tumors [64,65,66,67]. *TRERF1* gene encodes a basal cell cycle regulatory protein that has been shown to play a key role in development of estrogen independence/resistance in solid tumors—e.g., breast cancer [68]. *KDR* gene encoding a tyrosine kinase protein is necessary for survival, proliferation, and migration of endothelial cells [69], testis-specific vasculature [70], also being a potential target for antitumor therapy [71]. *MAP3K4* gene is an important component of the MAPK pathway, plays a crucial role in epithelial to mesenchymal transition, supports the male gonadal determination, and has been tested as a prognostic factor in other cancer types [72,73].

The signaling pathway analysis revealed an increased number of mutations in the cell cycle control and proteasome pathway in resistant NCCIT_CDDP cells, comparing with the other cell lines. This may underline the previously mentioned role of changes in DDR and cell cycle control, enabling the cell cycle arrest and repair of chemotherapy-induced adducts in the resistant cells.

TGCT are tumors with quite low TMB in comparison with other solid tumors of adults. This corresponds with the generally low amount of identified gene mutations among TGCT patients. Interestingly, the CDDP-resistant patient showed an increased TMB, contrary to the remaining patients.

In contrast to TMB, large CNVs are expected in TGCT and were also present in our sample cohort. As in the case of low TMB, the abundant CNVs are probably related to the TGCT origin. While the low amount of mutations detected in TGCT may be the result of embryonic DDR leading to apoptosis and not allowing for (aberrant) DNA repair, the germ cell character and abnormalities in cell divisions and mitotic/meiotic switch form strong predispositions for larger genome/chromosome rearrangements. The hallmark of TGCT—the amplification of the short arm of chromosome 12—was clearly present in all analyzed samples, both cell lines and patients. Of other CNV changes, several were present in the majority of samples, while additional ones were found in CDDP-resistant samples only. Of note, many of the newly acquired gains and losses in the resistant cell line and the resistant patient’s sample affected similar chromosomal loci. It again underlines the high functional significance of CNVs and larger genomic changes in TGCT development, only our understanding and interpretation of these very complex alterations is still limited.

## 4. Materials and Methods

### 4.1. Patients’ Samples

TGCT samples were obtained after patients’ informed consent and ethical committee (Thomayer Hospital and University Hospital in Motol) approval (latest amendments approval G-18-25 from 13 June 2018 and NV19-03-00302 from 20 June 2018, respectively). Their characteristics are summarized in Table 5.

### 4.2. Cell Lines

Two non-seminoma TGCT cell lines—NCCIT (Lot Number: 59069063) and Tera-2 (Lot Number: 4018167) were purchased from ATCC (American Type Culture Collection, Manassas, VA, USA) (details in Supplementary Material).

### 4.3. Treatment with Cisplatin

NCCIT and Tera-2 cells were treated with increasing concentrations of cisplatin (CDDP; Ebewe Pharma, Unterach am Attersee, Austria), as described in Supplementary Material.

### 4.4. Cell Cycle Assays and Proliferation Assay

To determine the differences in cell cycle distribution and cell proliferation between original and CDDP-treated cell lines, several staining protocols and measurements were used (details in Supplementary Material).

### 4.5. Nucleic Acid Isolation

Nucleic acids (DNA and RNA) from both the cell lines and patients’ frozen samples were isolated with DNeasy Mini Kit and RNeasy Mini Kit (Qiagen, Hilden, Germany). DNA and RNA from FFPE samples were extracted with RecoverAll Total Nucleic Acid Isolation Kit for FFPE (ThermoFisher Scientific) (details in Supplementary Material).

### 4.6. Whole Exome, Whole Transcriptome, and Amplicon Sequencing

Whole exome, amplicon and transcriptome sequencing were performed as described in Supplementary Material. Aberrations were visualized in Integrative Genome Viewer [74] (details in Supplementary Material).

The sequencing data are available at GEO, accession number GSE136560 (https://www.ncbi.nlm.nih.gov/geo/query/acc.cgi?acc=GSE136560).

### 4.7. Statistical Analysis

Data were analyzed using statistical software Excel (Microsoft Corporation, Redmond, WA, USA), Prism (GraphPad, La Jolla, CA, USA), and R-project [75].

The resistance of the cell lines to cisplatin, as determined by MTS assay, was assessed from the IC_50_ values, which were calculated in the R-project [75] using package drc [76] and further analyzed by Prism 7 (GraphPad Software).

All group comparisons were calculated using non-parametric tests (Mann–Whitney one tail test or Kruskall–Wallis test; GraphPad Software).

Principal component analysis was used to determine the genetic relations among the original and derived cell lines.

Kegg pathway analysis was made on WES data by counting and visualization of significant variants present in the selected group of pathways that could be assumed to be involved in TGCT development and progression.

## 5. Conclusions

In summary, our study suggests that the development of CDDP resistance in TGCT is associated with increased number of genetic aberrations on the level of SNVs/TMB as well as CNVs. Some of these aberrations may represent potential driver aberrations, while most of them may be the results of the on-going malignant transformation from a very unique type of tumor with specific embryonic and germ cell features to a malignant solid tumor with more general characteristics and higher genomic instability. Our findings support the hypothesis about the key role of a unique DDR for the high TGCT sensitivity to platinum-based chemotherapy; the acquired CDDP resistance being associated with decreased apoptotic response, changes in the cell cycle control with decreased proliferation and increased cell cycle arrest, allowing the repair of chemotherapy-induced damage. Several genes have been identified to have a potential effect on CDDP resistance development – some of them being previously referred to in relation to TGCT susceptibility (e.g., *DMRTA1*) or TGCT resistance (*MDM2*). The *ATRX* and *NSD1* genes, newly depicted in this study, represent strong candidates for the functional role in TGCT and CDDP resistance ethiopathology, though their effect will require further evaluation. Their identification also supports the emerging role of altered epigenetic gene regulation in TGCT biologic background.

## Figures and Tables

**Table 1:** Newly acquired variants detected by whole exome sequencing (WES) in CDDP-treated cells in comparison to the original cell lines.

Chr	Pos	Ref	Var	Var freq	Gene	Var Type	Function
**NCCIT_CDDP**							
chr13	114307689	G	T	94%	*ATP4B*	intron	P-type cation-transporting ATPase
chrX	76777763	G	T	95%	*ATRX*	missense	chromatin remodeling enzyme
chrX	76938527	C	A	100%	*ATRX*	missense	chromatin remodeling enzyme
chrX	76938528	C	A	100%	*ATRX*	missense	chromatin remodeling enzyme
chrX	39933413	C	A	89%	*BCOR*	missense	transcriptional corepressor
chrX	79980448	C	T	100%	*BRWD3*	missense	histone binding factor
chr19	18899229	T	A	84%	*COMP*	missense	extracellular matrix protein
chr4	101344524	AG	A	91%	*EMCN*	frameshift	mucin-like sialoglycoprotein
chr11	124794933	CG	C	81%	*HEPACAM*	frameshift	cell adhesion molecule
chr4	84240519	C	A	94%	*HPSE*	missense	remodeling of extracellular matrix
chr4	55984870	T	A	85%	*KDR*	missense	receptor tyrosine kinase
chr6	161455415	A	G	93%	*MAP3K4*	missense	MAPK kinase
chr4	170384486	C	CA	95%	*NEK1*	frameshift	serine/threonine kinase
chrX	101092554	G	T	88%	*NXF5*	missense	nuclear RNA export factor
chrX	38146319	C	T	98%	*RPGR*	missense	guanine nucleotide exchange factor
chr19	46299131	T	G	81%	*RSPH6A*	missense	unknown function in sperm cells
chr4	83788367	T	A	88%	*SEC31A*	missense	protein transporter
chr6	160679609	G	T	95%	*SLC22A2*	missense	cation transporter
chrX	69772065	C	A	92%	*TEX11*	missense	regulator of crossovers
chr4	6302537	G	C	98%	*WFS1*	missense	cation transporter
chr17	6673969	G	T	93%	*XAF1*	missense	regulator of apoptosis
chr7	50097636	G	T	83%	*ZPBP*	missense	zona pellucida binding protein
**Tera-2_CDDP**							
chrX	153418437	C	A	91%	*OPN1LW*	missense	red cone photopigment

Chr: chromosome, Pos: position, Ref: reference, Var: variant, Var freq: variant frequency, Var type: variant type.

**Table 2:** Tumor mutation burden in TGCT cell lines.

Cell Lines	Original [Variants/1 Mbp]	Derived [Variants/1 Mbp]	Ratio
NCCIT	139.5	152.5	1.09
Tera-2	143.8	152.3	1.06

**Table 3:** Somatic variants detected by WES in primary TGCT tumors (in comparison with germ-line control patient samples).

ID	Chr	Pos	Ref	Var	Var freq	Var Type	Gene	Function
pt2	chr5	94620080	G	T	33%	missense	*MCTP1*	cation transporter
pt2	chr5	156566309	A	T	32%	missense	*MED7*	transcription coactivator transcription
pt2	chr17	79650826	G	T	30%	missense	*ARL16*	cellular antiviral response
pt2	chr6	42227382	C	T	30%	missense	*TRERF1*	transcription factor
pt2	chr20	55206264	C	T	45%	missense	*TFAP2C*	transcription factor
pt2	chr7	45123888	C	T	41%	missense	*NACAD*	cellular antiviral response
pt3	chr19	3491580	G	T	30%	stop/gained	*DOHH*	metalloenzyme
pt4	chr3	42982835	G	T	38%	missense	*KRBOX1*	repressor of transcription
pt4	chr11	66101644	G	T	38%	missense	*RIN1*	RAS effector protein
pt4	chr11	121323287	G	T	38%	missense	*SORL1*	protein transporter
pt4	chr19	47259048	C	G	35%	missense	*FKRP*	ribitol-phosphate transferase
pt4	chr3	27762912	G	T	33%	missense	*EOMES*	transcription factor
pt4	chr9	22447573	G	T	30%	missense	*DMRTA1*	transcription factor
pt4	chr14	29236541	G	T	30%	missense	*FOXG1*	transcription factor

**Table 4:** Tumor mutation burden of WES-sequenced patients’ primary TGCT samples.

Patient	Control [Variants/1 Mbp]	Tumor [Variants/1 Mbp]	Ratio
pt1	383.5	647.9	1.69
pt2	107.9	459.0	4.25
pt3	162.5	401.7	2.47
pt4	170.2	490.8	2.88

**Table 5:** Patients’ characteristics.

ID	Age at Dg.	Histology	CS	Therapy	R	FUP [Months]	Survival at Last Control	Disease Status at Last Control
**WES**								
**pt1**	23	NS	IS	CHT		37	A	DF
**pt2**	29	NS	III	CHT		4	D	PD
**pt3**	38	NS	III	CHT+surg(RP LAE)	Y	42	A	DF
**pt4**	28	NS	IS	CHT		44	A	DF
**Amplicon seq**								
**A1**	46	S	IS	CHT		83	A	DF
**A2**	31	NS	IS	CHT		57	A	DF
**A3**	27	S	l	CHT		96	A	DF
**A4**	31	S	I	RT		60	A	DF
**A5**	23	NS	IS	CHT		81	A	DF
**A6**	22	S	lS	CHT		51	A	DF
**A7**	31	S	ll	CHT		138	A	DF
**A8**	42	NS	l	CHT		106	A	DF
**A9**	26	NS	I	CHT		52	A	DF
**A10**	44	NS	l	CHT		72	A	DF
**A11**	31	S	IS	RT		89	A	DF
**A12**	37	NS	l	CHT		46	A	DF
**A13**	37	S	I	RT		41	A	DF
**A14**	35	NS	III	CHT		57	A	DF
**A15**	43	S	II	CHT		28	A	DF
**A16**	29	S	l	CHT		37	A	DF
**A17**	23	NS	II	CHT		72	A	DF
**A18**	29	S	l	RT		39	A	DF
**A19**	48	NS	IS	CHT		92	A	DF
**A20**	58	S	l	CHT		16	A	DF
**A21**	30	NS	IS	CHT		19	A	DF
**A22**	34	S	II	CHT		20	A	DF
**A23**	65	NS	III	CHT	Y	11	D	PD
**A24**	33	S	I	RT		23	A	DF
**A25**	40	NS	l	CHT		27	A	DF
**A26**	21	NS	IS	CHT		23	A	DF
**A27**	37	S	l	CHT		24	A	DF

WES: pts analyzed by whole exome sequencing, Amplicon seq: pts analyzed by amplicon sequencing, NS: nonseminoma, S: seminoma, CS: clinical stage, R: relapse, CHT: chemotherapy, RT: radiotherapy, surg (RP LAE): surgery (retroperitoneal lymfadenectomy), Y: yes, FUP: follow-up, A: alive, D: dead, DF: disease-free, PD: progressive disease.

## Supplementary Materials

Supplementary material and figures are available online at https://www.mdpi.com/2072-6694/11/9/1316/s1. Figure S1: Keqq pathway analysis of SNPs detected in CDDP-naive and CDDP-treated cell lines, Figure S2: Scattered plots displaying CNVs of TGCT cell lines inferred from WES data normalized to pooled control samples, Figure S3: Number of SNPs present in at least 20% of sequencing reads in FFPE TGCT samples (analyzed by amplicon sequencing) depending on the tumor clinical stage or histologic type, Figure S4: Scattered plots displaying CNVs of TGCT primary tumor samples inferred from WES data normalized to pooled control samples, Table S1: Fusion genes detected in NCCIT and Tera-2 cell lines by transcriptome sequencing.
