# Supplementary material for: Molecular Basis of Cisplatin Resistance in Testicular Germ Cell Tumors
Source: Cancers (Basel). 2019 Sep 6;11(9):1316. doi: 10.3390/cancers11091316 (PMC6769617; doi:10.3390/cancers11091316)
Supplement: Supplementary file 1 [file cancers-11-01316-s001.pdf]

Supplementary Materials

Molecular Basis of Cisplatin Resistance in Testicular Germ Cell Tumors

Violeta Bakardjieva-Mihaylova, Karolina Skvarova Kramarzova, Martina Slamova, Michael Svaton, Katerina Rejlova, Marketa Zaliova, Alena Dobiasova, Karel Fiser, Jan Stuchly, Marek Grega, Blanka Rosova, Roman Zachoval, Petr Klezl, Vaclav Eis, Eva Kindlova, Tomas Buchler, Jan Trka and Ludmila Boublikova

Methods

Cell Lines

No seminoma cell line was available from the official cell-line collections. For the experiments we thus used T-Cam cell line donated to our department but due to the later uncertainties regarding its origin and characteristics, the results obtained on this cell line were finally excluded from the analyses and statistics.

All cell lines were cultured in RPMI 1640 medium supplemented with 10% fetal bovine serum (FBS) and penicillin/streptomycin (all from ThermoFisher Scientific, Waltham, MA, USA) at 37 °C in a humidified cell culture incubator with 5% carbon dioxide. Prior to passaging experiments, the cells were washed with PBS and trypsinized with 0.25% Trypsin in phosphate buffered saline (PBS).

Treatment with Cisplatin

NCCIT and Tera-2 cells were treated with increasing concentrations of cisplatin (CDDP; Ebewe Pharma, Unterach am Attersee, Austria) starting with the concentration of 0.1 μM. When the lethality of the cells reached 80%, the cells were left to recover over four passages without CDDP. The final concentration of CDDP used for establishment of resistant cell lines was 2 μM for NCCIT and only 0.1 μM for Tera-2 cell line. The same cell lines were also long-term cultivated without CDDP to detect aberrations that occur spontaneously due to prolonged cultivation and distinguish them from those induced by CDDP treatment.

Resistance to cisplatin was assessed from IC_{50} as determined by MTS proliferation assay performed repeatedly every 3 months. For every cell line, the analysis was performed in parallel with CDDP-treated and -untreated cells. The final data were collected from 3 independent experiments in case of Tera-2 and from 4 independent experiments in case of NCCIT (each performed at least in 3 replicates). The cells were incubated with serial concentrations of CDDP for 72 hours and their proliferation/metabolic activity was measured by Cell Titer 96® Aqueous One Solution Cell Proliferation Assay (Promega, Madison, WI, USA).

The proliferation and cell cycle assays were performed with the established cell lines when cultured in media without the presence of CDDP; the apoptotic assays were performed both without CDDP and with CDDP (10 μM) added to the culture medium. The analyses were performed in triplicates.

Cell Proliferation Assay

To determine the proliferation rate of TGCT cell lines, we assessed the total number of cells arising from the equal initial number of seeded cells over the period of two weeks. Measurements were carried out repeatedly in triplicates.

Cell Cycle Assays

EdU Staining

The cell lines were cultured for 1.5 hours with 10 μM EdU (Click-iT® Plus EdU Imaging Kits, ThermoFisher Scientific). After centrifugation, the cells were treated with 10% formaldehyde for 10 minutes at room temperature and 0.1% Triton for another 15 minutes at 37 °C. The cells were then washed with 4% FBS in PBS, spun down and stained with Alexa fluor 647 at room temperature for 30 minutes. Proliferation of the cells was measured using LSR II instrument (BD Biosciences, Franklin Lakes, NJ, USA).

Pyronin/Hoechst Staining

After resuspension in phosphate-citrate buffer solution (pH 4.8), the cells were incubated at room temperature for 20 minutes in the dark, spun down, washed repeatedly with PBS, spun down, washed once with 1 ml staining solution (PBS with 1.5 μg/mL pyronin Y and 2 μg/mL Hoechst 33342), spun down and incubated with 200 μL of the same staining solution for 10 minutes on ice. Cell cycle analysis was performed using LSR II instrument (BD Biosciences). All centrifugation steps were performed in the same way (1000 rpm, 5 min).

Apoptosis Assay

Apoptosis rate of the cells was in all cell lines determined without any treatment and after 72 hours of treatment with 10 μM CDDP. The cells were stained with annexin V-Dy647 (RCANXD-T100, Exbio, Vestec, Czech Republic) and propidium iodide (PI) solution (130-093-233, Miltenyi Biotec, Bergisch Gladbach, Germany). The proportion of apoptotic and necrotic cells was measured using LSR II instrument (BD Biosciences).

Nucleic Acid Isolation

DNA from the cell lines and patients´ frozen samples was isolated with DNeasy Mini Kit (Qiagen, Hilden, Germany); RNA from the cell lines and patients´ frozen samples was isolated with RNeasy Mini Kit (Qiagen, Hilden, Germany). DNA and RNA from FFPE samples were extracted with RecoverAll Total Nucleic Acid Isolation Kit for FFPE (ThermoFisher Scientific). Concentration, quality, and integrity of nucleic acids were evaluated by spectrophotometry—Nanodrop 2000 and Qubit 2.0 Fluorometer (both ThermoFisher Scientific), capillary electrophoresis—Agilent 2100 Bioanalyzer (Agilent Technologies, Santa Clara, CA, USA) and by PCR. Synthesis of cDNA was performed using the iScript kit (Bio-Rad, Hercules, CA, USA) starting from 1 μg of total RNA. All procedures were carried out according to the manufacturers' instructions.

Whole Exome Sequencing

Exome libraries from cell line DNA samples were prepared using SureSelectXT Human All Exon V5+UTR kit (Agilent Technologies) according to the manufacturer instruction, with starting amount of 50 ng of DNA. The samples were sequenced on NextSeq 500 Instrument (Illumina, San Diego, CA, USA) using a High Output Kit v2 (150 cycles) (Illumina) according to the manufacturer's instructions. Libraries from patients' samples were prepared using Nimblegen SeqCap EZ Human Exome Library v2.0 kit (Roche, Basel, Switzerland) starting with 1 μg of gDNA and sequencing was performed on HiSeq 2500 (Illumina) in Atlas Biolabs (Berlin, Germany). The paired-end sequencing was employed ($n \geq 1.6 \times 10^8$ of paired reads) with median Phred quality score above 30. On average, 92% reads mapped to target regions (minimum 72%) with mean coverage 50×.

Whole Transcriptome Sequencing

Whole transcriptome sequencing was performed with RNA isolated from the TGCT cell lines. Transcriptome libraries were prepared using the SureSelectXT RNA Reagent Kit (Agilent Technologies), and carried out on NextSeq500 (Illumina) by using a High Output Kit according to the

manufacturer's instruction. Mean coverage was 121.44 (minimum 76.43) with duplication rate below 60%.

Amplicon Sequencing

DNA from FFPE samples of TGCT patients was subjected to amplicon sequencing using Ion AmpliSeq Comprehensive Cancer Panel (ThermoFisher Scientific) with all-exon coverage of over 400 genes involved in cancer pathogenesis. The libraries were prepared with Ion AmpliSeq Library Kit 2.0 and 200 bp chemistry (Ion PI Template OT2 200 kit v2 and Ion One Touch 2 System). The samples were sequenced using Ion PI Sequencing 200 Kit v2, Ion PI Chip v2 and the Ion Proton Sequencer platform (all ThermoFisher Scientific, Waltham, MA, USA) performed as described in [27]. All procedures were carried out according to the manufacturer's instructions and detailed protocols are available on manufacturer's websites.

Sequencing Data Analysis

The detected sequences were separated from the technical noise, their quality was checked (FastQC, QualiMap), they were aligned to the reference genome sequence (hg19) (BWA,)[77] and the variants were called (VarScan2 [78], samtools [79]). The alterations detected were further analyzed for their potential clinical effect (Ingenuity), annotated by SnpEff [80] and matched with the publically available databases, genome atlases and other on-line sources (The Cancer Genome Atlas, UCSC, ONCOMINE, CLINVAR). Aberrations were visualized in Integrative Genome Viewer [74], and checked manually for their presence (both strand reads), absence in control samples, frequency, quality and sequence context (e.g., a presence of homopolymers, etc.).

For WES sequencing, only significant variants that passed the filtration criteria were further evaluated (absolute quality reads number ≥5, variant allele ratio in control samples (germ-line or platinum sensitive) ≤0.03, variant allele ratio in studied primary tumor samples ≥0.3, in platinum treated cell lines ≥0.80, synonymous variants excluded). The identified variants were confirmed to be unique for cisplatin-resistant cells by checking their low/no presence in the CDDP-naive long-term in parallel cultured cell lines.

Similarly, significant variants reported in case of amplicon sequencing of primary tumor samples were filtered in if variant allele ratio was ≥0.2 in tumor samples and ≤0.03 in germline controls; variant effect was defined as nonsense, missense, stop/loss, frameshift, and unknown; and variant clinical effect defined as pathogenic, probably pathogenic, drug response, other and unknown.

Tumor mutation burden (TMB) was calculated as a number of called single nucleotide variants (SNVs) filtered in (with absolute quality reads number ≥5, variant frequency ≥0.05, synonymous variants excluded), related to the length of exons covered.

Copy number variants were predicted from WES data using CNV kit [81] with normalization to pooled control samples sequenced on the same instrument using identical library preparation kits. Predicted segmental changes were calculated for all samples and segments with predicted CN different from 2 were selected for further comparison. Selected regions were plotted in heatmap diagrams for all samples.

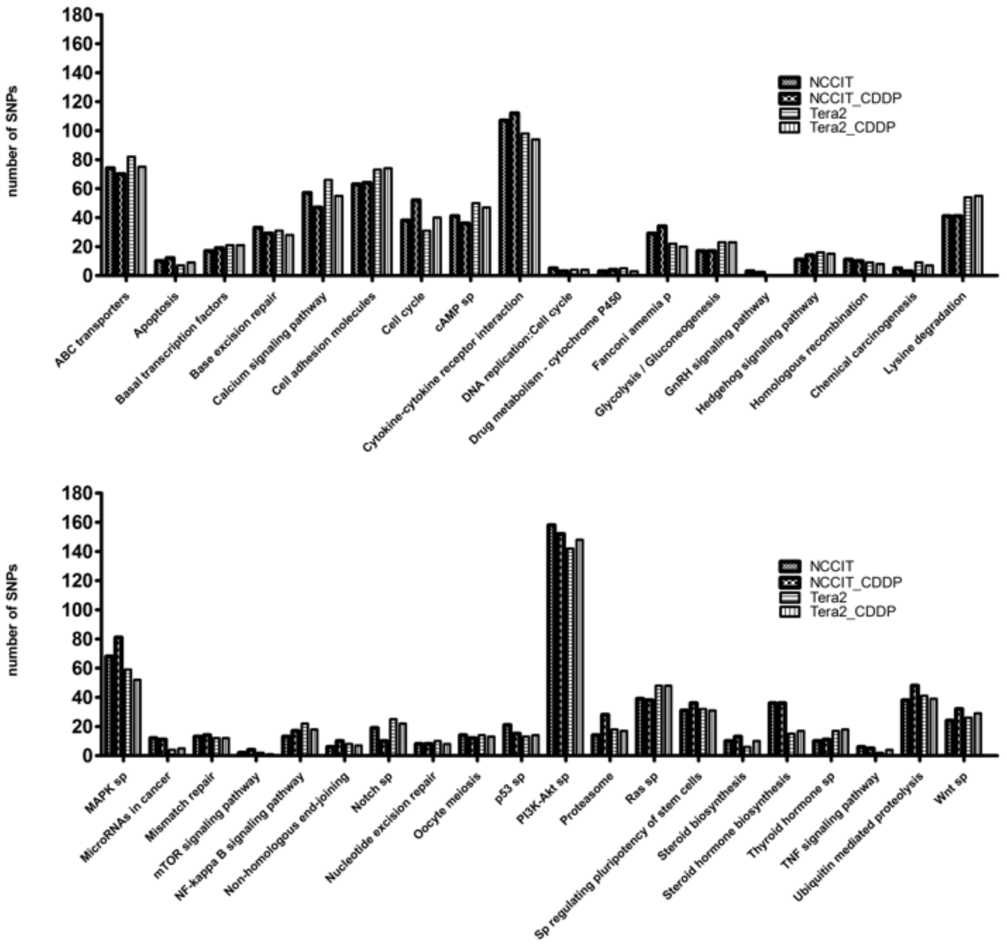

Figure S1. Keqq pathway analysis of SNPs detected in CDDP-naive and CDDP-treated cell lines.

Table S1. Fusion genes detected in NCCIT and Tera-2 cell lines by transcriptome sequencing.

Cell line	Gene 1	Gene2	Position 1	Position 2	Origin
**NCCIT**					
	SBF2	*RNF141*	chr2(42472827)	chr2(42836598)	in-frame deletion
	EML4	*MTA3*	chr11(10215449)	chr11(10546920)	amplification (tandem duplication)
**Tera-2**					
	SPG7	*CDH15*	chr16 (89614520)	chr16 (89251580)	amplification (tandem duplication)
	ZNHIT6	*COL24A*	chr1 (86167839)	chr1 (86315088)	amplification (tandem duplication)
	ZNF160	*ZNF415*	chr19 (53606517)	chr19 (53613160)	amplification (tandem duplication)

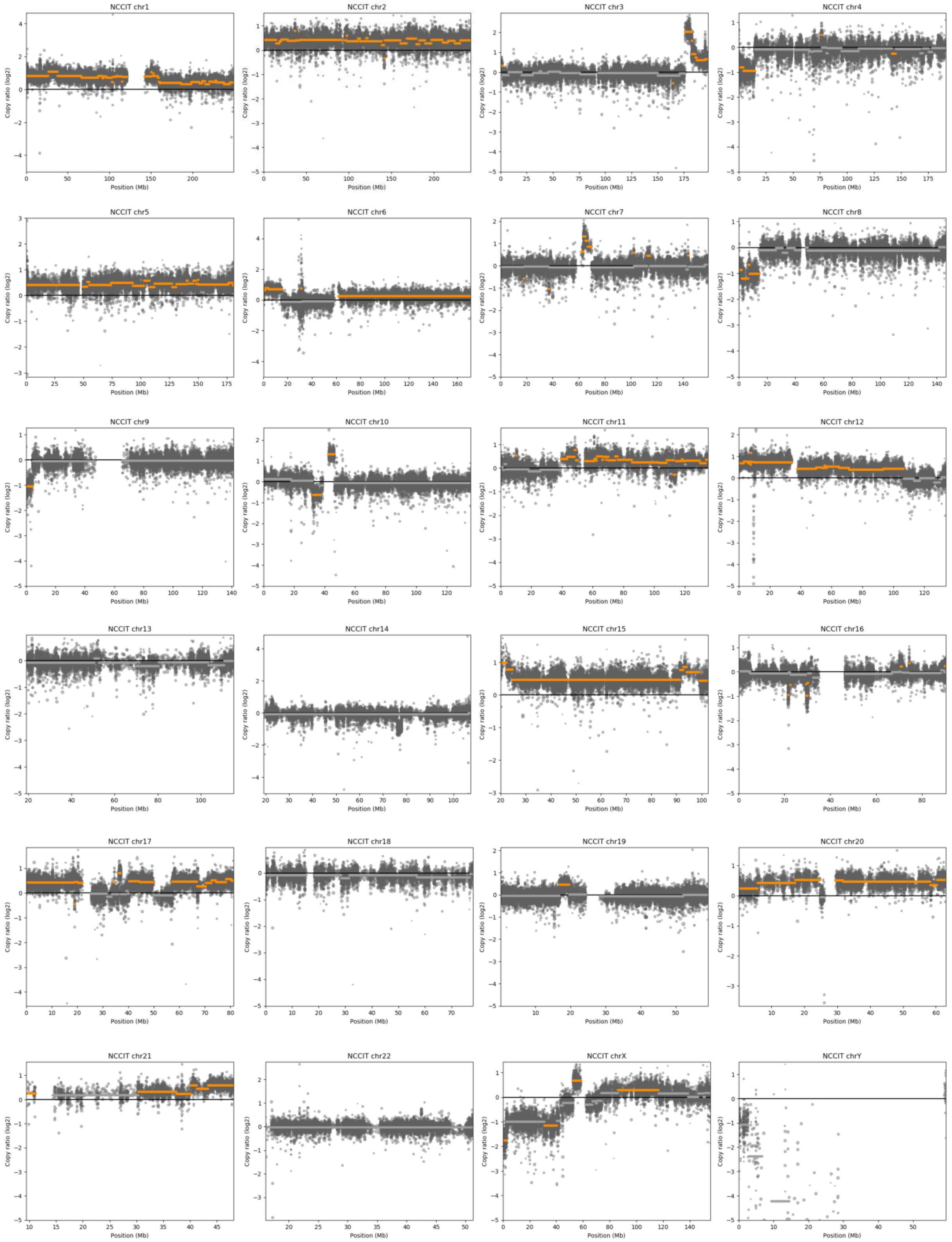
NCCIT chr1
NCCIT chr2
NCCIT chr3
NCCIT chr4
NCCIT chr5
NCCIT chr6
NCCIT chr7
NCCIT chr8
NCCIT chr9
NCCIT chr10
NCCIT chr11
NCCIT chr12
NCCIT chr13
NCCIT chr14
NCCIT chr15
NCCIT chr16
NCCIT chr17
NCCIT chr18
NCCIT chr19
NCCIT chr20
NCCIT chr21
NCCIT chr22
NCCIT chrX
NCCIT chrY
Copy ratio (log2)
Position (Mb)

(a)

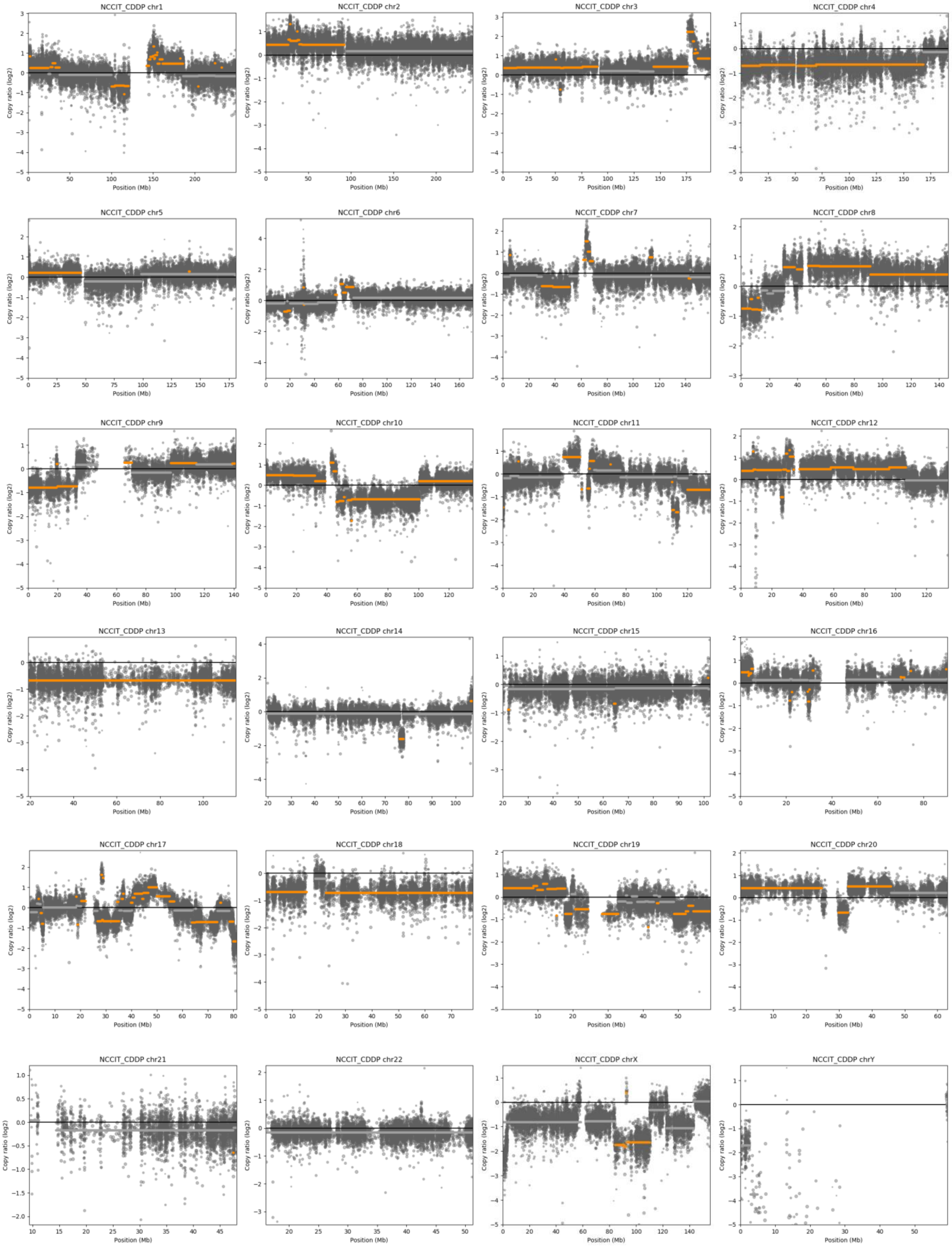
NCCIT_CDDP chr1
NCCIT_CDDP chr2
NCCIT_CDDP chr3
NCCIT_CDDP chr4
NCCIT_CDDP chr5
NCCIT_CDDP chr6
NCCIT_CDDP chr7
NCCIT_CDDP chr8
NCCIT_CDDP chr9
NCCIT_CDDP chr10
NCCIT_CDDP chr11
NCCIT_CDDP chr12
NCCIT_CDDP chr13
NCCIT_CDDP chr14
NCCIT_CDDP chr15
NCCIT_CDDP chr16
NCCIT_CDDP chr17
NCCIT_CDDP chr18
NCCIT_CDDP chr19
NCCIT_CDDP chr20
NCCIT_CDDP chr21
NCCIT_CDDP chr22
NCCIT_CDDP chrX
NCCIT_CDDP chrY
Copy ratio (log2)
Position (Mb)

(b)

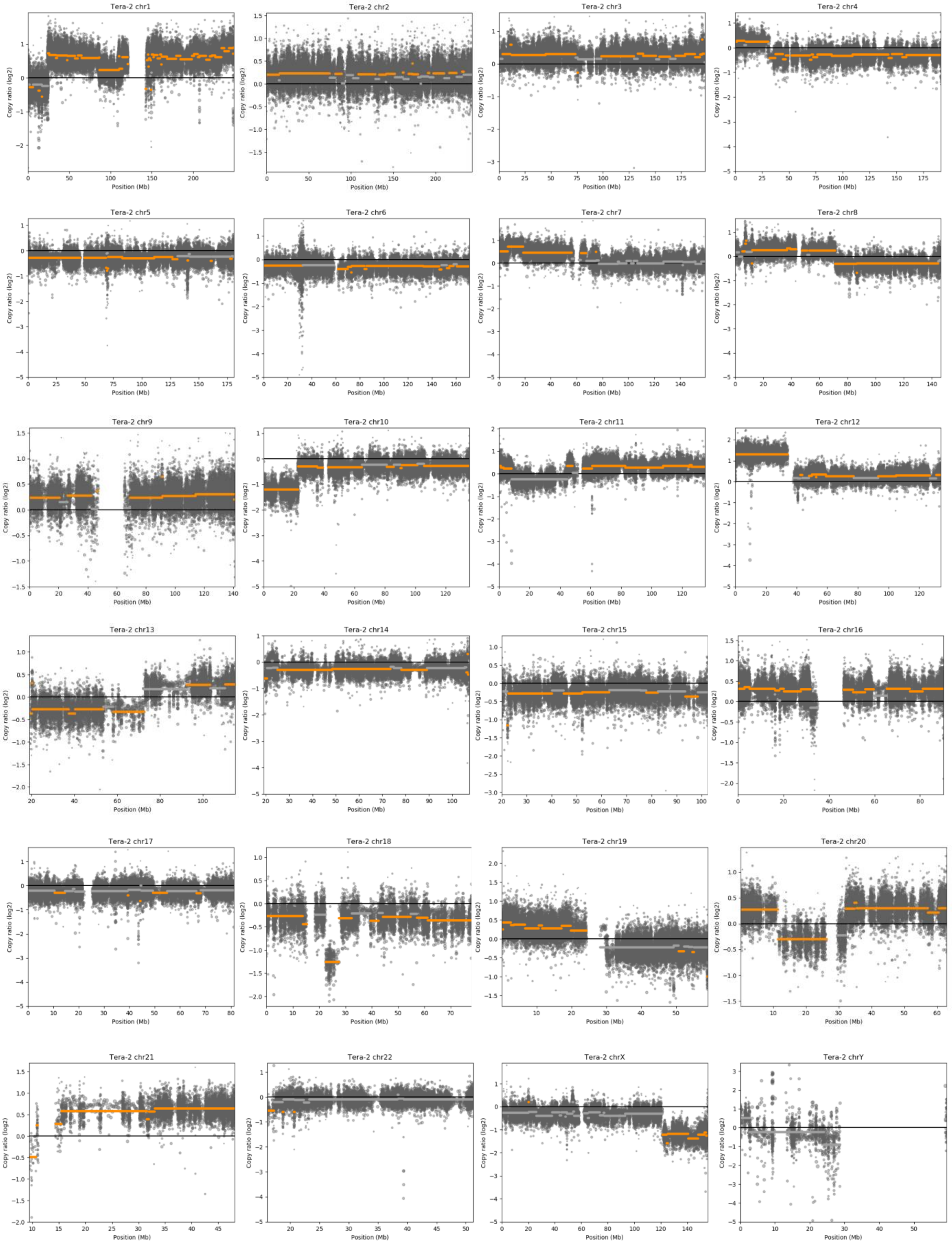
Tera-2 chr1
Tera-2 chr2
Tera-2 chr3
Tera-2 chr4
Tera-2 chr5
Tera-2 chr6
Tera-2 chr7
Tera-2 chr8
Tera-2 chr9
Tera-2 chr10
Tera-2 chr11
Tera-2 chr12
Tera-2 chr13
Tera-2 chr14
Tera-2 chr15
Tera-2 chr16
Tera-2 chr17
Tera-2 chr18
Tera-2 chr19
Tera-2 chr20
Tera-2 chr21
Tera-2 chr22
Tera-2 chrX
Tera-2 chrY
Copy ratio (log2)
Position (Mb)

(c)

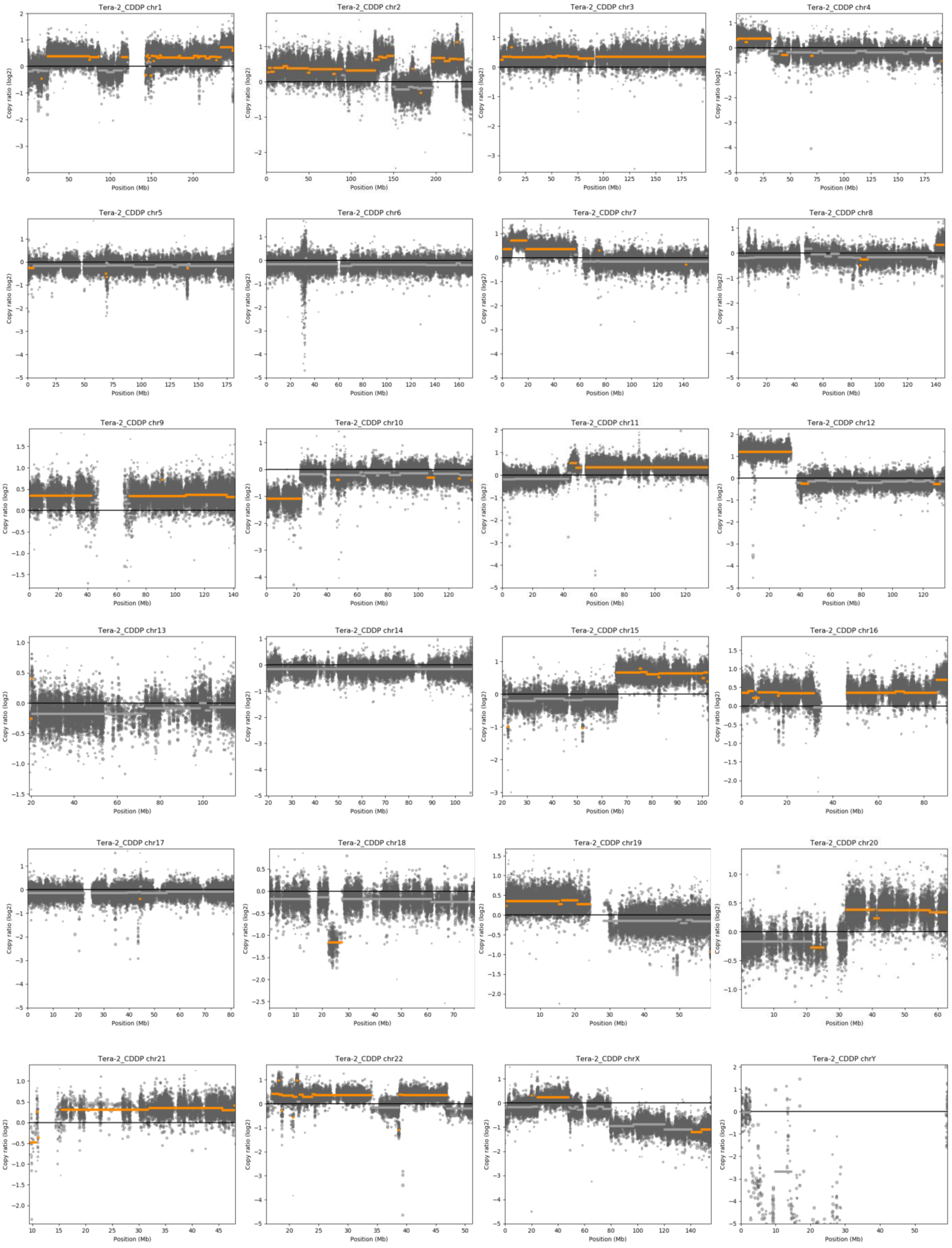

(d)

Figure S2. Scattered plots displaying CNVs of TGCT cell lines inferred from WES data normalized to pooled control samples: (**a**) NCCIT, (**b**) NCCIT_CDDP, (**c**) Tera-2, (**d**) Tera-2_CDDP. Segmental changes with predicted copy number alteration are marked in orange, normal in gray.

Correlation of the number of SNPs with clinical stage or histologic type of the tumor

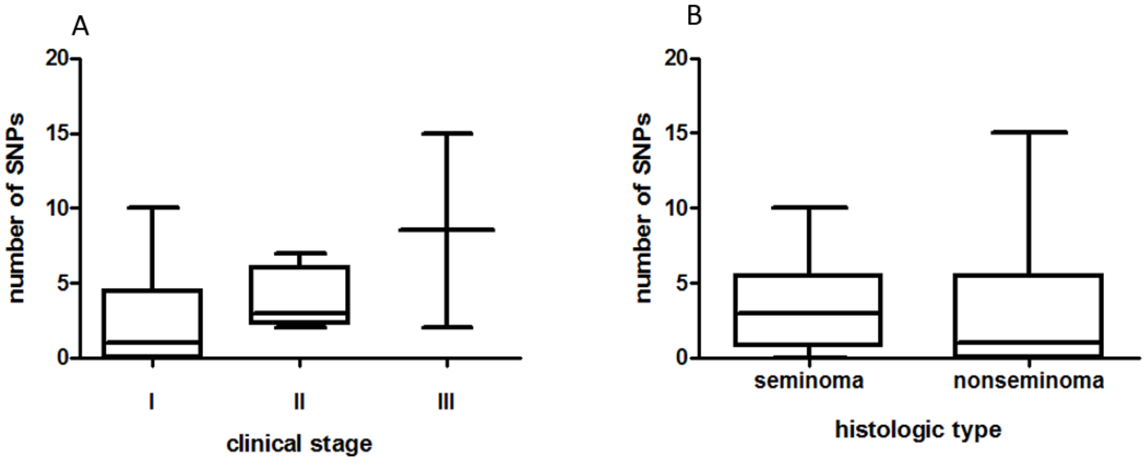

Figure S3. Number of SNPs present in at least 20% of sequencing reads in FFPE TGCT samples (analyzed by amplicon sequencing) depending on the tumor clinical stage or histologic type.

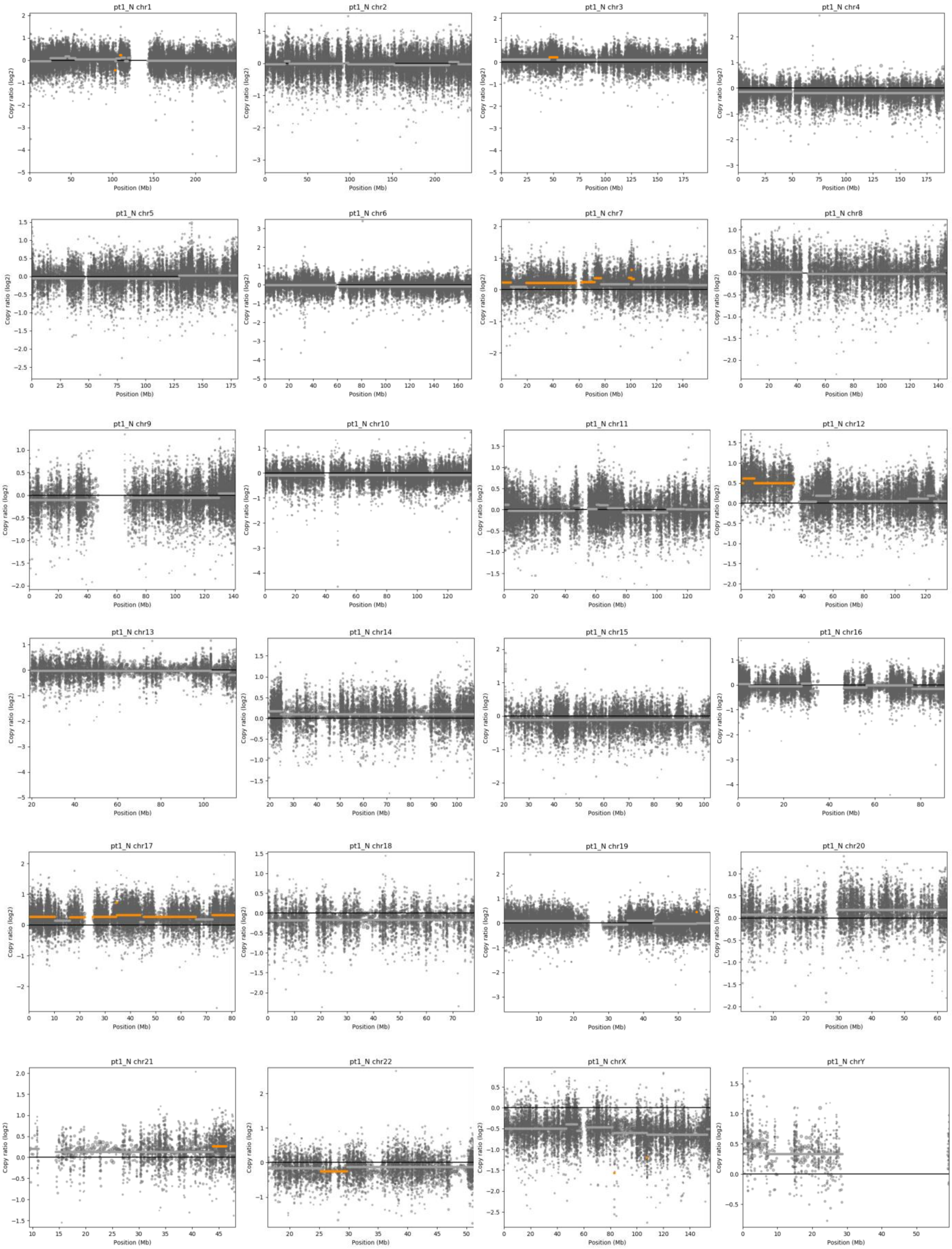
pt1_N chr1
pt1_N chr2
pt1_N chr3
pt1_N chr4
pt1_N chr5
pt1_N chr6
pt1_N chr7
pt1_N chr8
pt1_N chr9
pt1_N chr10
pt1_N chr11
pt1_N chr12
pt1_N chr13
pt1_N chr14
pt1_N chr15
pt1_N chr16
pt1_N chr17
pt1_N chr18
pt1_N chr19
pt1_N chr20
pt1_N chr21
pt1_N chr22
pt1_N chrX
pt1_N chrY
Copy ratio (log2)
Position (Mb)

(a)

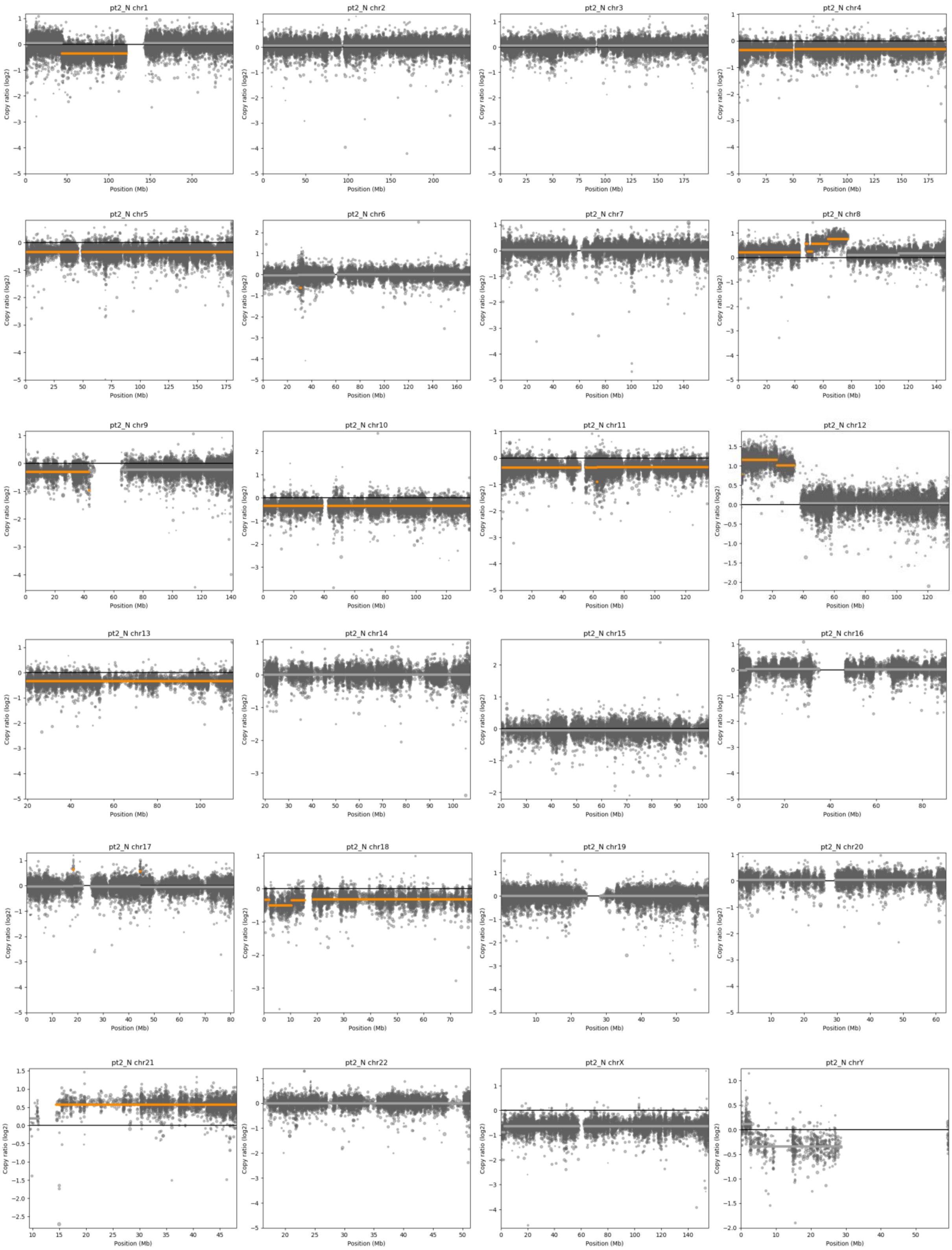
pt2_N chr1
pt2_N chr2
pt2_N chr3
pt2_N chr4
pt2_N chr5
pt2_N chr6
pt2_N chr7
pt2_N chr8
pt2_N chr9
pt2_N chr10
pt2_N chr11
pt2_N chr12
pt2_N chr13
pt2_N chr14
pt2_N chr15
pt2_N chr16
pt2_N chr17
pt2_N chr18
pt2_N chr19
pt2_N chr20
pt2_N chr21
pt2_N chr22
pt2_N chrX
pt2_N chrY
Copy ratio (log2)
Position (Mb)

(b)

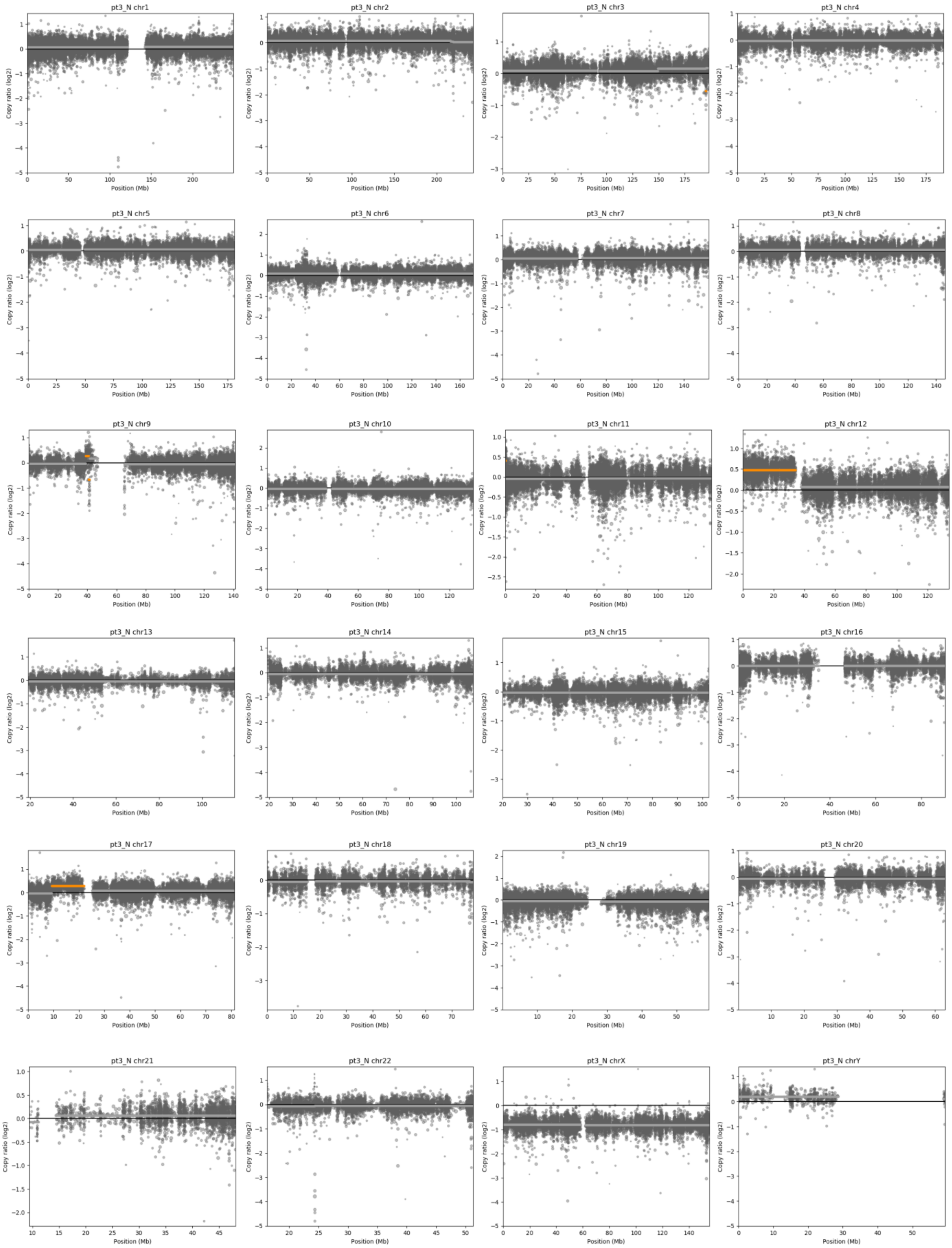
pt3_N chr1
pt3_N chr2
pt3_N chr3
pt3_N chr4
pt3_N chr5
pt3_N chr6
pt3_N chr7
pt3_N chr8
pt3_N chr9
pt3_N chr10
pt3_N chr11
pt3_N chr12
pt3_N chr13
pt3_N chr14
pt3_N chr15
pt3_N chr16
pt3_N chr17
pt3_N chr18
pt3_N chr19
pt3_N chr20
pt3_N chr21
pt3_N chr22
pt3_N chrX
pt3_N chrY
Copy ratio (log2)
Position (Mb)

(c)

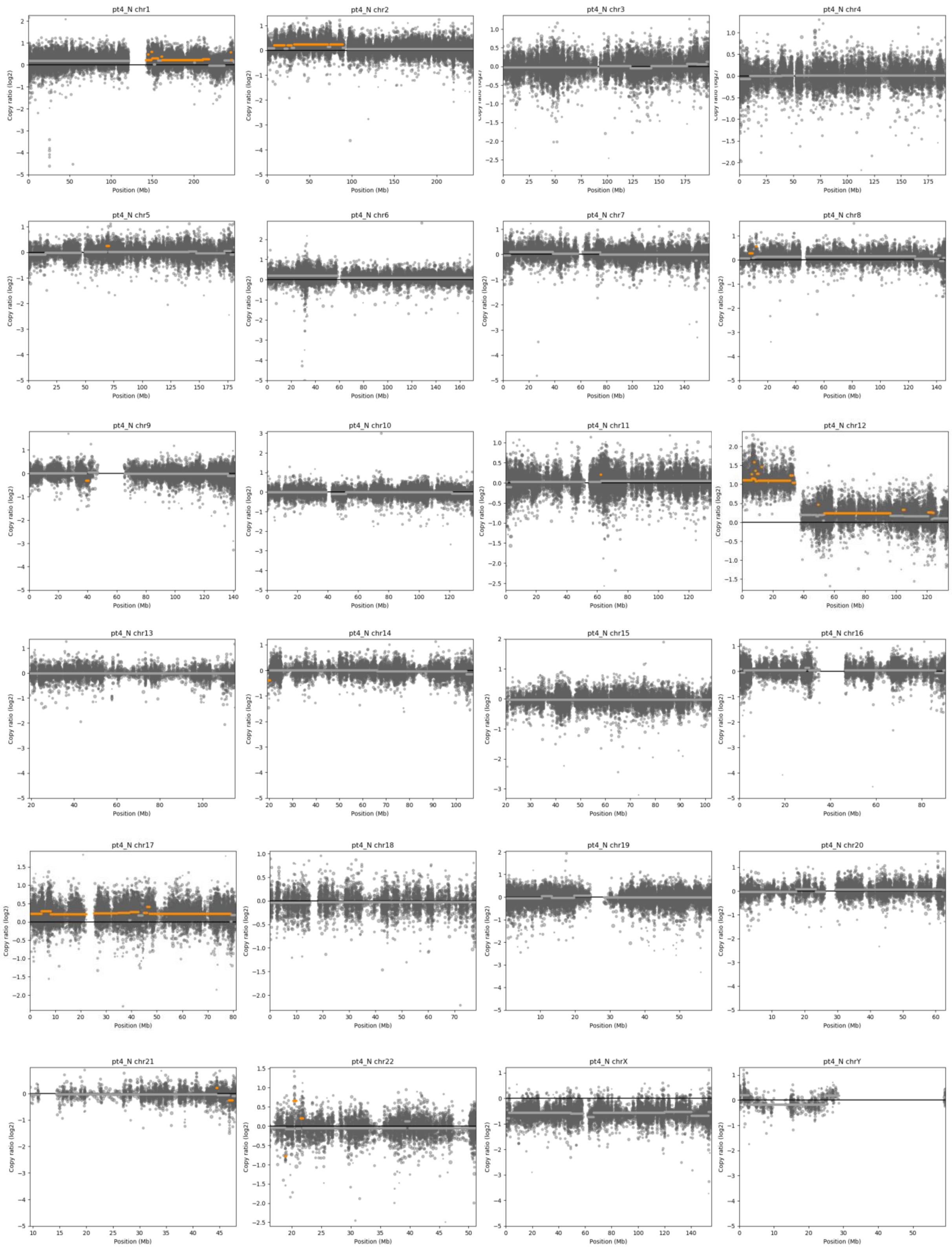

(d)

Figure S4. Scattered plots displaying CNVs of TGCT primary tumor samples inferred from WES data normalized to pooled control samples: (**a**) pt1, (**b**) pt2, (**c**) pt3, (**d**) pt4. Segmental changes with predicted copy number alteration are marked in orange, normal in gray.

References

77. Li, H.; Durbin, R. Fast and accurate long-read alignment with Burrows-Wheeler transform. *Bioinformatics* **2010**, *26*, 589–595.

78. Koboldt, D.C.; Zhang, Q.; Larson, D.E.; Shen, D.; McLellan, M.D.; Lin, L.; Miller, C.A.; Mardis, E.R.; Ding, L.; Wilson, R.K. VarScan 2: somatic mutation and copy number alteration discovery in cancer by exome sequencing. *Genome Res.* **2012**, *22*, 568–576.
79. Li, H.; Handsaker, B.; Wysoker, A.; Fennell, T.; Ruan, J.; Homer, N.; Marth, G.; Abecasis, G.; Durbin, R.; 1000 Genome Project Data Processing Subgroup The Sequence Alignment/Map format and SAMtools. *Bioinformatics* **2009**, *25*, 2078–2079.
80. Cingolani, P.; Patel, V.M.; Coon, M.; Nguyen, T.; Land, S.J.; Ruden, D.M.; Lu, X. Using Drosophila melanogaster as a Model for Genotoxic Chemical Mutational Studies with a New Program, SnpSift. *Front. Genet.* **2012**, *3*.
81. Talevich, E.; Shain, A.H.; Botton, T.; Bastian, B.C. CNVkit: Genome-Wide Copy Number Detection and Visualization from Targeted DNA Sequencing. *PLoS Comput. Biol.* **2016**, *12*, e1004873.

© 2019 by the authors. Licensee MDPI, Basel, Switzerland. This article is an open access article distributed under the terms and conditions of the Creative Commons Attribution (CC BY) license (http://creativecommons.org/licenses/by/4.0/).
